# High frequencies of circulating memory T cells specific for calreticulin exon 9 mutations in healthy individuals

**DOI:** 10.1038/s41408-018-0166-4

**Published:** 2019-01-17

**Authors:** Morten O. Holmström, Shamaila M. Ahmad, Uffe Klausen, Simone K. Bendtsen, Evelina Martinenaite, Caroline H. Riley, Inge M. Svane, Lasse Kjær, Vibe Skov, Christina Ellervik, Niels Pallisgaard, Hans C. Hasselbalch, Mads H. Andersen

**Affiliations:** 1 Department of Hematology, Zealand University Hospital, Roskilde, Denmark; 2 Center for Cancer Immune Therapy, Department of Hematology, Copenhagen University Hospital Herlev, Herlev, Denmark; 3 Department of Hematology, Rigshospitalet, Copenhagen, Denmark; 4 Department of Oncology, Copenhagen University Hospital Herlev, Herlev, Denmark; 5 Faculty of Health and Medical Sciences, University of Copenhagen, Copenhagen, Denmark; 6 Department of Production, Research, and Innovation, Region Zealand, Sorø, Denmark; 7 Department of Laboratory Medicine, Boston Children’s Hospital, Harvard Medical School, Boston, MA USA; 8 Department of Surgical Pathology, Zealand University Hospital, Roskilde, Denmark; 9 Department of Immunology and Microbiology, University of Copenhagen, Copenhagen, Denmark

## Abstract

Mutations in exon 9 of the calreticulin gene (*CALR*) frequently occur in patients with chronic myeloproliferative neoplasms (MPN). Patients exhibit spontaneous cellular immune responses to epitopes derived from the mutant CALR C-terminus, and *CALR*-mutant-specific T cells recognize autologous *CALR*-mutant malignant cells. This study investigated whether *CALR*-mutant-specific T cells occur naturally in *CALR*wt MPN-patients and in healthy individuals. Specific immune responses against epitopes in the mutant CALR peptide sequence were detected in both *CALR*wt MPN-patients and in healthy individuals. Healthy donors displayed more frequent and stronger CALR-mutant specific T-cell responses compared to the responses identified in *CALR*-mutant MPN-patients. Several T-cell responses were identified in healthy donors directly ex vivo. Importantly, by running functional analyses on live-sorted immune cells from healthy donors, we showed that circulating *CALR*-mutant-specific immune cells are T-memory cells. These findings suggest, that healthy individuals acquire a *CALR* exon 9 mutation, but the immune system reacts and clears the mutant cells, and during this reaction generates *CALR*-mutant specific T-memory cells. We believe that these findings provide the evidence for tumor immune surveillance in MPN.

## Introduction

In 2013, two independent research groups reported on the occurrence of somatic mutations in exon 9 of the calreticulin (*CALR*) gene in 75% of patients with Janus kinase 2 (*JAK2*)wt chronic myeloproliferative neoplasms (MPN)^1,2^. We recently provided evidence that patients with *CALR*-mutant MPN exhibit frequent, spontaneous cellular immune responses against epitopes derived from the mutant CALR C-terminus^3^, and that *CALR*-mutant-specific T-cells derived from patients can recognize and kill autologous *CALR*-mutant cells in a *CALR*-mutant dependent manner^4^. As such the *CALR* mutations generate an immunogenic antigen that could be used as target for cancer immune therapy^5^.

As the *CALR* mutations are only identified in patients with myeloid cancer, the immune system in healthy individuals should not have been challenged with mutant CALR epitopes, and hence should not react to stimulation with these. Accordingly, several studies on immune reactivity against other shared commonly occurring mutations, such as *pRAS* mutations and *BRAF* mutations in solid cancers, have indicated that cells from healthy individuals fail to show spontaneous immune responses against these neoantigens^6,7^. In regard to immune responses to the BCR-ABL fusion transcript, one study showed responses in peripheral blood mononuclear cells (PBMC) from 3/18 healthy donors after stimulation in vitro with autologous dendritic cells that had been pulsed with a BCR-ABL peptide^8^, whereas another study failed to show immune responses against the transcript in healthy individuals^9^. Other studies have focused on immune responses against the somatic *MYD88*L265P mutation, which occurs in several types of lymphoma^10^. One study demonstrated a T-cell response, when T-cells derived from healthy individuals were stimulated with the *MYD88*L265P epitope, but the responses required two stimulations with peptide-pulsed dendritic cells^11^.

Of interest, none of the above studies have demonstrated immune responses against tumor-specific antigens in cells from healthy individuals tested in an ex vivo setting, without prior in vitro peptide stimulation. Even more, it has never been shown, which cells in the T-cell compartment actually harbor these neo-antigen-specific T cells.

Given the high immunogenicity of the *CALR* exon 9 mutations we investigated if healthy donors display T-cell responses specific for the *CALR* mutations and if so, whether such CALR-mutant specific T cells are antigen experienced T-memory cells (T_mem_) or naive T cells (T_naive_). The identification of a memory response is important, as CALR-mutant specific T cells in the T_mem_ compartment suggest that healthy donors may acquire a *CALR* exon 9 mutation, which is cleared by specific T-cells and T_mem_ is established in the process.

This study demonstrates that healthy donors display stronger and more frequent CALR-mutant specific T-cell responses compared to *CALR*-mutant patients. Even more, we show that the mutant CALR C-terminus harbors an immunogenic hotspot, and finally that CALR-mutant specific T cells in healthy individuals are T_mem_.

## Materials and methods

### Materials

Buffy coats from anonymized blood donors were acquired from the blood bank at Rigshospitalet, Copenhagen, Denmark. Peripheral blood mononuclear cells (PBMC) from patients with *JAK2*V617F-mutant MPN were acquired after informed consent from the patient. PBMC were isolated with Lymphoprep (Axis Shield, Oslo, Norway) and frozen in fetal calf serum with 10% dimethyl sulfoxide (Sigma-Aldrich, St. Louis, MO, USA). To determine whether age might influence specific immune responses to *CALR*-mutants, we isolated PBMCs from 50 healthy individuals of different ages. All participants provided informed consent, in agreement with the Helsinki Declaration, before study entry. The project was approved by the local Ethics Committee of the Zealand Region (Approval number SJ-175 and SJ-456).

### Peptides

**Fig. 1:**
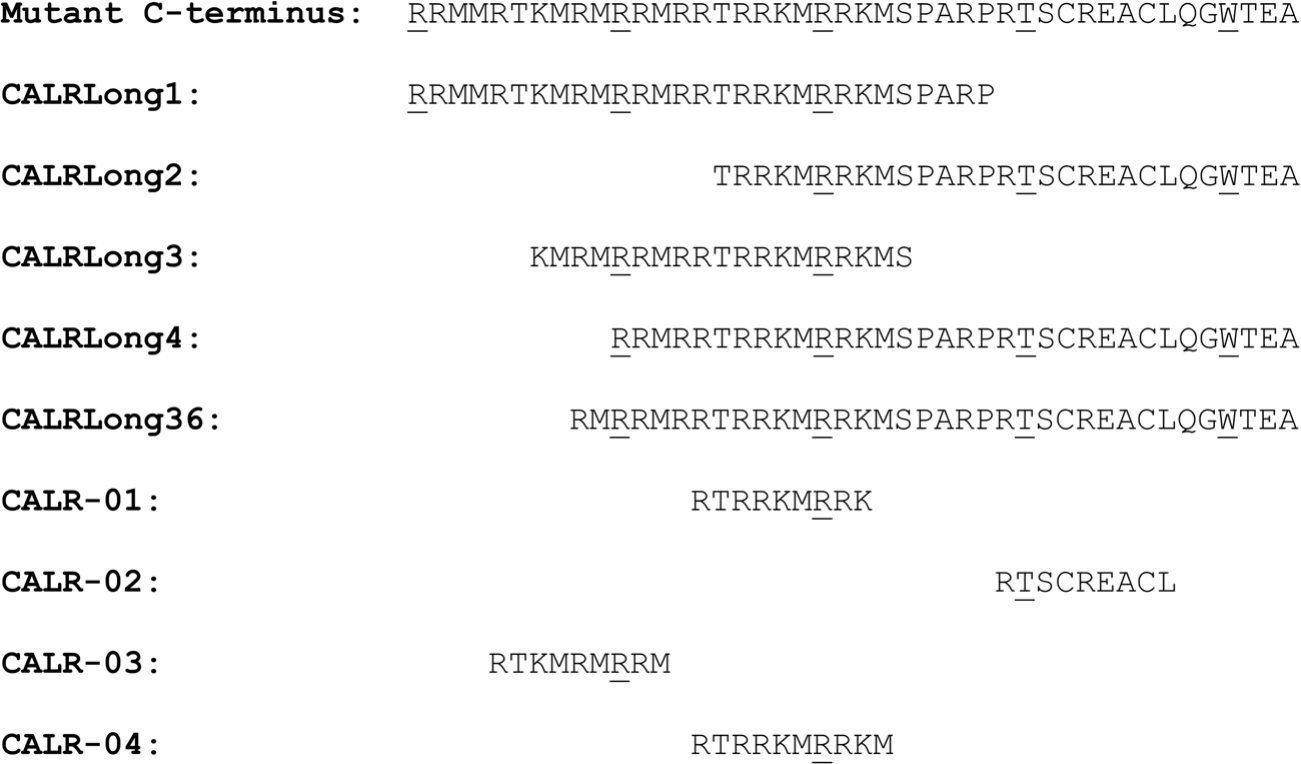
Amino acid sequences for the long and short epitopes used in this study. The sequences are shown relative to the mutant CALR C-terminus. Every 10th amino acid is underscored to facilitate tracking

We chose to work with the following peptides, which were either provided by KJ Ross-Petersen (Klampenborg, Denmark) or Schafer-N (Copenhagen, Denmark): CALRLong1 (RRMMRTKMRMRRMRRTRRKMRRKMSPARP), CALRLong2 (TRRKMRRKMSPARPRTSCREACLQGWTEA), CALRLong3 (KMRMRRMRRTRRKMRRKMS), and CALRLong4 (RRMRRTRRKMRRKMSPARPRTSCREACLQGWTEA). In addition, we used the CALRLong36 (RMRRMRRTRRKMRRKMSPARPRTSCREACLQGWTEA) peptide, provided by PolyPeptide Laboratories (Strasbourg, France). We also chose to work with the following short peptides, all provided by KJ Ross-Petersen (Klampenborg, Denmark): CALR-01 (RTRRKMRRK), CALR-02 (RTSCREACL), CALR-03 (RTKMRMRRM), and CALR-04 (RTRRKMRRKM). The sequences of peptides and their relation to the entire CALR-mutant C-terminal are given in Fig. 1. To identify the immunogenic hotspot in the mutant CALR C-terminus, we segregated the entire mutant C-terminus into nonamer epitopes, with eight overlapping amino acids. Thus, we generated a total of 36 nonamer peptides, which we termed the CALR library (Supplementary Material 1). These epitopes were acquired from Pepscan (Lelystad, Netherlands). Negative control peptides were generated by scrambling the CALR-mutant peptide sequence. We generated three negative control peptides with the following sequences: MRRTMMMMMPRRRRRRKRRSKTRAPRMRK, MERKMAEQRPCRKPSTRALATCRRWGSRT, and TSMMRRRRRRKRRKMMKRM. These peptides were provided by KJ Ross-Petersen.

### Enzyme-linked immunospot assay

To analyze *CALR*-mutant-specific immune responses, we employed Enzyme-Linked ImmunoSPOT (ELISPOT) assays, according to protocols described previously^12^. Immune responses were analyzed with the interferon-gamma (IFN-γ) or tumor necrosis factor alpha (TNF-α) ELISPOT assay. Unless otherwise stated, all experiments were performed with the in vitro IFN-γ ELISPOT assay. The ELISPOT assays employed 1.3 to 4 × 10^5^ cells/well. Unless otherwise stated, all ELISPOT experiments were performed in triplicates. Additionally, when not otherwise stated, ex vivo ELISPOTs were performed with thawed PBMCs that had been allowed to rest overnight at 37 °C in an incubator with 5% CO_2_ and a humidified atmosphere. ELISPOT experiments were set-up as previously described^12^. ELISPOT plates were analyzed and counted using the ImmunoSpot S6 Ultimate Analyzer (CTL Analyzers, Shaker Heights, OH, USA).

For the identification of immune responses against epitopes in the CALR library, we deviated from the standard in vitro stimulation protocol. For cultures that we planned to analyze for responses against the B1-C6 region of the CALR-mutant C-terminus, we performed in vitro stimulations with the CALRLong1 peptide. For cultures that we planned to analyze for response against the C7-D12 region of the CALR-mutant C-terminus, we performed in vitro stimulation with the CALRLong2 peptide. The in vitro assays with CALRLong1 and CALRLong2 saved time and cells, with minimal effects on the sensitivity of the experiments, because CALRLong1 spanned residues B1-C6, and CALRLong2 spanned residues C7-D12.

### Magnetically activated cell sorting (MACS)

CD4^+^ T_mem_ cells and T_naive_ were sorted with the MACS method, with the Memory CD4^+^ T-cell Isolation Kit (order no 130–091–893) and with the Naive Pan T-cell Isolation Kit (order no 130–097–095), respectively (both Miltenyi Biotech, Bergish Gladbach, Germany), according to the manufacturer’s protocols. Cells were thawed and rested overnight, then sorted the next day. Cells were sorted twice on a magnetic column to ensure high purity in the enriched cells. During T_naive_ isolation, we added the optional anti-TCR γ/δ antibodies to ensure depletion of γ/δ T cells. We analyzed the purity of the enriched fractions by staining with Fixable Viability Stain 510, CD3-APC-H7, CD45RO-PE, and CD45RA-APC (all BD Biosciences, San José, CA, USA). Cells were analyzed on a FACS Canto II flow cytometer (BD Biosciences, San José, CA, USA) equipped with FACS Diva Software. Gating strategy for the identification of CD3^+^ T cells for the phenotypic analysis of memory cell markers is provided in Supplementary Material 2.

### Fluorescence-activated cell sorting (FACS) of live cells

Cells were thawed, and then rested overnight in x-vivo medium (Lonza, Basel, Switzerland) with 5% human serum in an incubator. The next day, cells were washed twice in FACS buffer, then stained with the following: LIVE/DEAD Fixable Near-IR Dead Cell Stain Kit (Waltham, MA, USA), CD45RA-APC, CD62L-PE, and CD3-FITC (BD Biosciences, San José, CA, USA). Cells were stained for 30 min on ice, washed twice, and then resuspended in FACS buffer at 50 × 10^6 ^cells/mL. Next, cells were sorted on a FACS ARIA flow cytometer with appropriate application settings and compensation controls. Cell sorting was performed with a purity setting. After sorting, cells were allowed to rest in x-vivo medium (Lonza, Basel, Switzerland) in an incubator. After sorting of all the cells, samples of 10^4^ cells were analyzed to ensure proper sorting purity. Gating strategy for the identification of CD3^+^ T cells for the phenotypic analysis and subsequent sorting of different memory cell fractions is provided in Supplementary Material 2. Materials and methods for intracellular cytokine staining (ICS), establishment of T-cell cultures specific for mutant CALR epitopes and statistical analyses are provided in Supplementary Material 3.

## Results

### Patients with *JAK2*V617F-mutant MPN harbor an immune response to mutant CALR epitopes

**Fig. 2:**
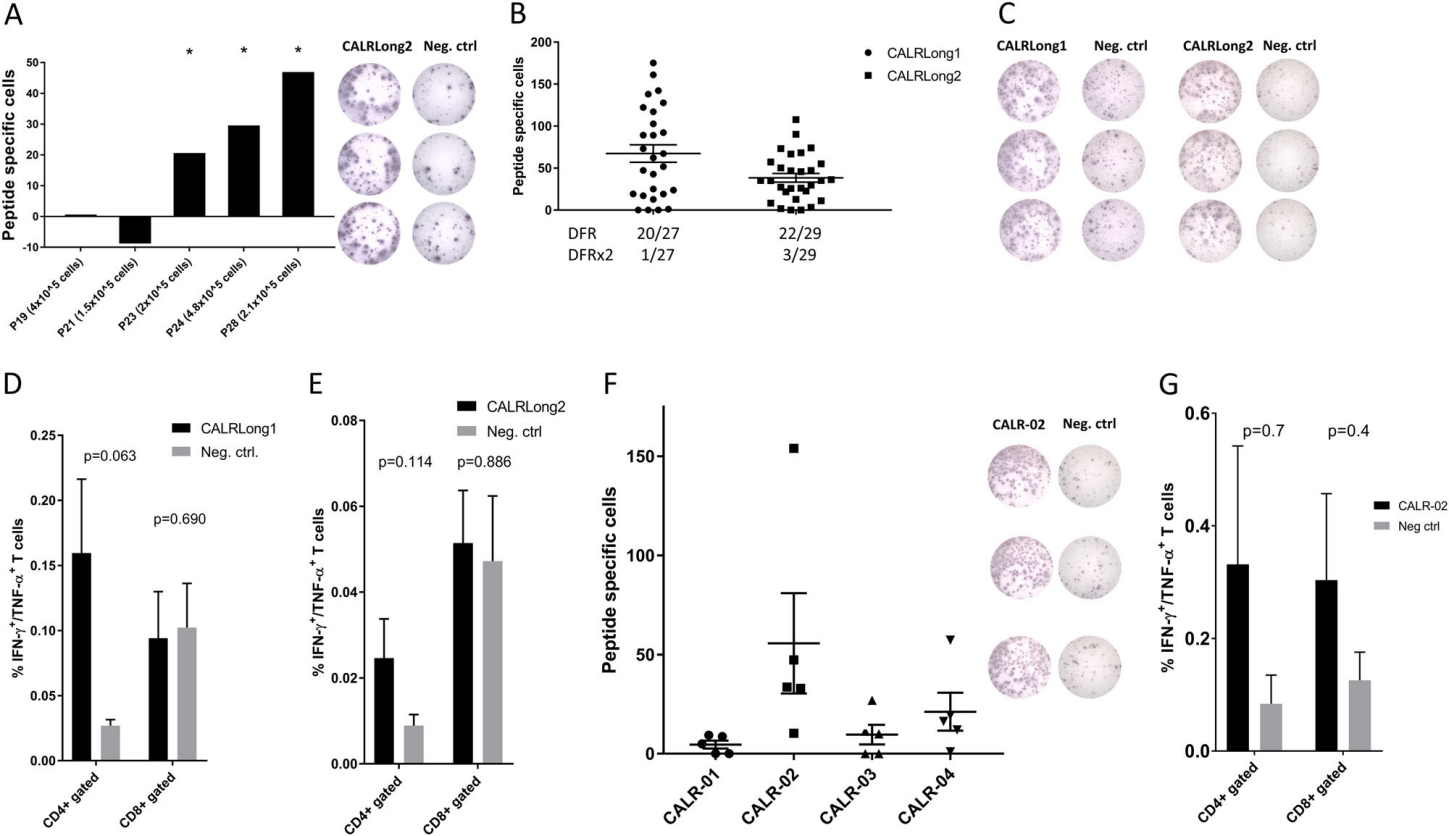
Spontaneous CD4^+^ and CD8^+^ T-cell responses against several epitopes in the mutant CALR C-terminus in healthy donors. **a** Cells from five patients with *JAK2*V617F-mutant MPN were analyzed for responses against CALRLong2 in an IFN-γ ELISPOT with varying effector cell concentrations. (*Left*) The number of spot-forming cells in control wells was subtracted from the number of spot-forming cells in peptide-stimulated wells. (*Right*) Example of an ELISPOT performed with PBMCs from one patient that was *JAK2*V617F^+^; PBMCs were stimulated with or without the CALRLong2 peptide. **b** The numbers of healthy individuals that showed specific immune responses to CALRLong1 and CALRLong2 peptides in IFN-γ ELISPOT. DFR-defined and DFR2x-defined responses are indicated. **c** Example of wells from healthy individuals that responded to (*left*) CALRLong1 and (*right*) CALRLong2. **d**, **e** Intracellular cytokine staining performed in cells from healthy individuals shows that CD4^+^ T-cells were double-positive for TNF-α and IFN-γ, after stimulation with either: **d** CALRLong1 (*n* = 5), or **e** CALRLong2 (*n* = 4), compared to a negative control scrambled peptide. **f** (*Left*) Cells from five healthy individuals were analyzed for spontaneous immune responses against three nonamer epitopes (CALR-01, CALR-02, CALR-03) and one decamer epitope derived from the CALR-mutant C-terminus. No samples showed DFR-defined responses against CALR-01; 4/5 samples responded to CALR-02; 1/5 samples responded to CALR-03; and 1/5 samples responded to CALR-04. (*Right*) Example of a response against CALR-02. **g** Cells from three healthy individuals that showed immune responses against CALR-02. Intracellular staining shows TNF-α and IFN-γ production in CD4^+^ and CD8^+^ T-cells stimulated with CALRLong2, compared to unstimulated cells. Error bars display standard error of the mean. *p ≤ 0.05 according to the DFR rule

We scrutinized peripheral blood mononuclear cells (PBMC) from patients with *JAK2*V617F-mutant polycythemia vera (PV) for immune responses against the immunogenic CALRLong1 and CALRLong2 epitopes that are derived from the mutant CALR C-terminus^3^. Surprisingly, 2/6 patients with PV exhibited a significant response against CALRLong1 (data not shown) and 3/5 had a significant immune response against CALRLong2 (Fig. 2a), according to the distribution free resampling method (DFR)^13^. *CALR*/*JAK2* double mutants are very rare and these mutations are generally mutually exclusive^14–17^.

### Cells from healthy individuals display strong and frequent immune responses against the CALRLong1 and CALRLong2 peptides and against short CALR-mutant epitopes

Next, we investigated whether cells from healthy individuals might also exhibit an immune response against the CALRLong1 and CALRLong2 epitopes. Surprisingly, cells from 20/27 (74%) individuals showed a significant response to CALRLong1 (Fig. 2b, c), and cells from 22/29 (76%) individuals showed a significant immune response to CALRLong2 (Fig. 2b, c). Using ICS, 5 and 4 healthy individuals were analyzed for responses against CALRLong1 (Fig. 2d) and CALRLong2 (Fig. 2e), respectively. Stimulation of PBMC with mutant CALR epitopes lead to increased production of TNF-α and IFN-γ from CD4^+^ T-cells. The responses were only borderline significant, probably due to low sample size.

Given these highly surprising results, we investigated the immunogenic potential of three CALR-mutant nonamer epitopes (CALR-01, CALR-02, and CALR-03) and one CALR-mutant decamer epitope (CALR-04). CALR-02 induced responses in cells from 4/5 individuals, and CALR-03 and CALR-04 induced responses in cells from one individual each (Fig. 2f). Furthermore, we tested cytokine release in three donors against CALR-02 using ICS (Fig. 2g). However, the difference in cytokine release between stimulated and unstimulated cells did not reach statistical significance.

### Screening for immune responses against nonamer epitopes in the mutant CALR C-terminus identifies an immunogenic hotspot sequence

Given the remarkably high frequency of responses against the CALR-mutant epitopes we verified that there was no homology between the 44 amino acid mutant CALR sequence and other known epitopes by the Basic Local Alignment Search Tool (BLAST)^18^ using the *blastpprogramme* and the non-redundant protein sequences (nr) database. We next examined whether the CALR-mutant specific immune responses might be directed towards a certain part of the mutant sequence. Hence, we divided the 44-amino acid mutant C-terminus that is shared between the majority of CALR-mutant patients, into nonamer epitopes, with eight overlapping amino acids (Supplementary Material 1). Accordingly, we generated 36 nonamer epitopes, and analyzed PBMCs from ten healthy individuals for immune responses against each of these epitopes. We observed immune responses against all parts of the mutant CALR sequence (Supplementary Material 4); however, we could clearly identify an immunogenic hotspot located in the B6 to C7 region. Thus, although all parts of the mutant CALR C-terminus were immunogenic, the most immunogenic part (the hotspot) was located in the second quartile of the mutant C-terminus.

### Cells from healthy subjects display strong, frequent immune responses against peptides spanning the entire mutant CALR C-terminus

**Fig. 3:**
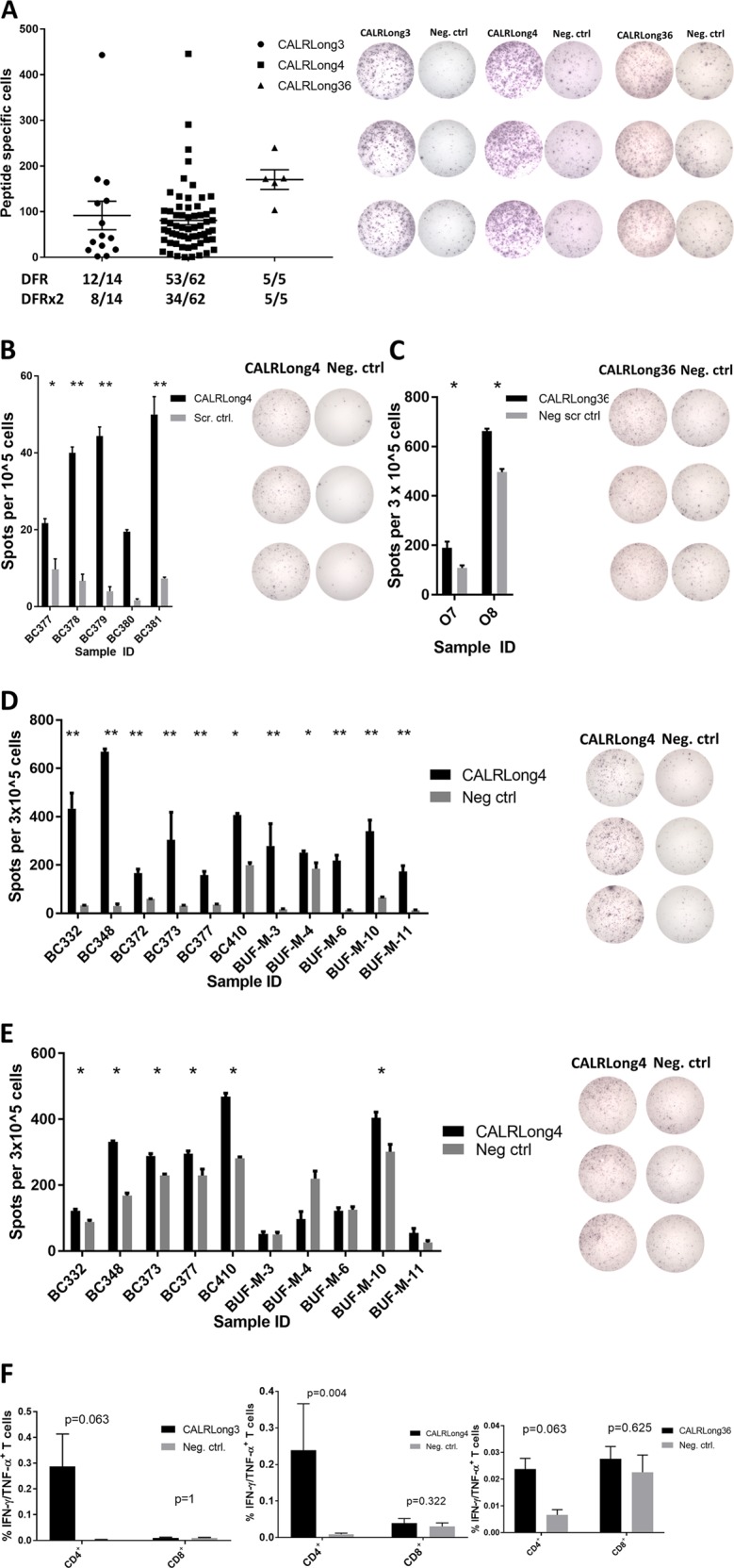
In vitro and ex vivo immune responses against epitopes that spanned the entire mutant C-terminus. **a** (*Left*) In vitro IFN-γ ELISPOT results show the number of cells that specifically responded to CALRLong3, CALRLong4, and CALRLong36 peptides. The DFR-defined and DFR2x-defined responses are indicated below the graph. Example of responses against CALRLong3 (*left*), CALRLong4 (*middle*), and CALRLong36 (*right*). **b** (*Left*) Ex vivo 22 h IFN-γ ELISPOT responses against CALRLong4 in cells from five healthy individuals; (*right*) example of a response. **c** (*Left*) Ex vivo 22 h IFN-γ ELISPOT responses against CALRLong36 in cells from two healthy individuals. **d** Ex vivo 72 h IFN-γ ELISPOT against CALRLong4 in cells from 11 healthy individuals, with wells displaying a representative response shown to the right. **e** Ex vivo 72 h TNF-α ELISPOT against CALRLong4 in cells from ten healthy individuals, with wells displaying a representative response shown to the right. **f** Intracellular cytokine staining of cells from healthy individuals depicts IFN-γ/TNF-α-double-positive CD4^+^ T-cells stimulated with either CALRLong3 (*left*), CALRLong4 (*middle*), or CALRLong36 (*right*). Cells from ten healthy donors were analyzed for responses against CALRLong4, and cells from five healthy donors were analyzed for response against CALRLong3 and CALRLong5. Error bars display standard error of the mean. *p ≤ 0.05 according to the DFR rule. **p ≤ 0.05 according to the DFR2x rule

As the B7-C6 hotspot sequence seemed to be highly immunogenic we merged the sequence into one long peptide (CALRLong3) and analyzed the immunogenicity of this epitope. Not surprisingly, 12/14 healthy donors harbored a response to CALRLong3 (Fig. 3a). However, our analysis of the CALR library showed that immune responses are identified agains all parts of the C-terminus. Therefore, we analyzed immune responses against CALRLong4, which spans the 34 most C-terminal amino acids in the mutant C-terminus, and CALRLong36, that spans all 36 amino acids in the CALR-mutant C-terminus. The immunogenicity of the latter was of particular interest, as this peptide is used in the phase I clinical vaccination trial currently running at our institution (NCT03566446). Both CALRLong4 and CALRLong36 incited frequent and strong responses (Fig. 3a). We then performed ELISPOT assays on PBMC plated directly ex vivo and allowed to incubate in the ELISPOT plate for 22 h. Ex vivo responses against CALRLong4 was found in 4/5 analyzed samples, and three samples displayed a DFR2x-defined significant response (Fig. 3b). Likewise, 2/2 analyzed samples showed an ex vivo response against CALRLong36 (Fig. 3c). As the CALRLong4 and CALRLong36 peptides are long peptides and, therefore, need antigen processing for presentation on the cell surface, the 22 h ex vivo ELISPOT may not show the full response to the mutant epitopes. As such, we performed 72 h ex vivo IFN-γ ELISPOT in PBMC from 11 healthy donors, (Fig. 3d) and TNF-α ELISPOT in PBMC from ten healthy donors (Fig. 3e). All 11 donors had an IFN-γ response, and six displayed a TNF-α response, once more demonstrating that the CALR-mutant epitopes are highly immunogenic, and the responses identified are indeed elicited by circulating CALR-mutant-specific T-cells. By using ICS on in vitro stimulated cultures to investigate the phenotype of the cytokine-producing cells stimulated with CALRLong3, CALRLong4 and CALRLong36 we found that it is mainly CD4^+^ T-cells that are activated upon antigen stimulation (Fig. 3f).

### The frequency of CALR-mutant-specific immune responses does not change with age

As the median age of diagnosis of *CALR*-mutant MPN-patients is 47–57 years^16,19–22^, we wondered if the emergence of *CALR*-mutant MPN could be explained by an age dependent loss of CALR-mutant specific immune response. Hence, we analyzed the specific responses against CALRLong1 and CALRLong4 in PBMC from 25 healthy individuals with a median age of 19 years (range 18–21 years) and in PBMC from 25 healthy individuals with a median age of 57 years (range 50–64 years). No difference in frequency nor amplitude of responses against CALRLong1 and CALRLong4 was identified between the two age defined cohorts (Supplementary Material 5A, 5B).

In total we have analyzed the CALR- mutant specific immune responses in a vast amount of healthy subjects: Of 74 subjects analyzed for response against CALRLong1, 54 (78%) exhibited a DFR-defined significant immune response, and 24 (32%) exhibited a DFR2x-defined significant response. The responses against CALRLong4 were even more frequent; of 110 subjects analyzed, PBMC from 98 (89%) exhibited a DFR-defined significant response and 60 individuals (55%) exhibited a DFR2x-defined significant response.

### CALR-mutant specific immune responses are identified in CD4^+^ T_mem_, but not T_naive_ cells

**Fig. 4:**
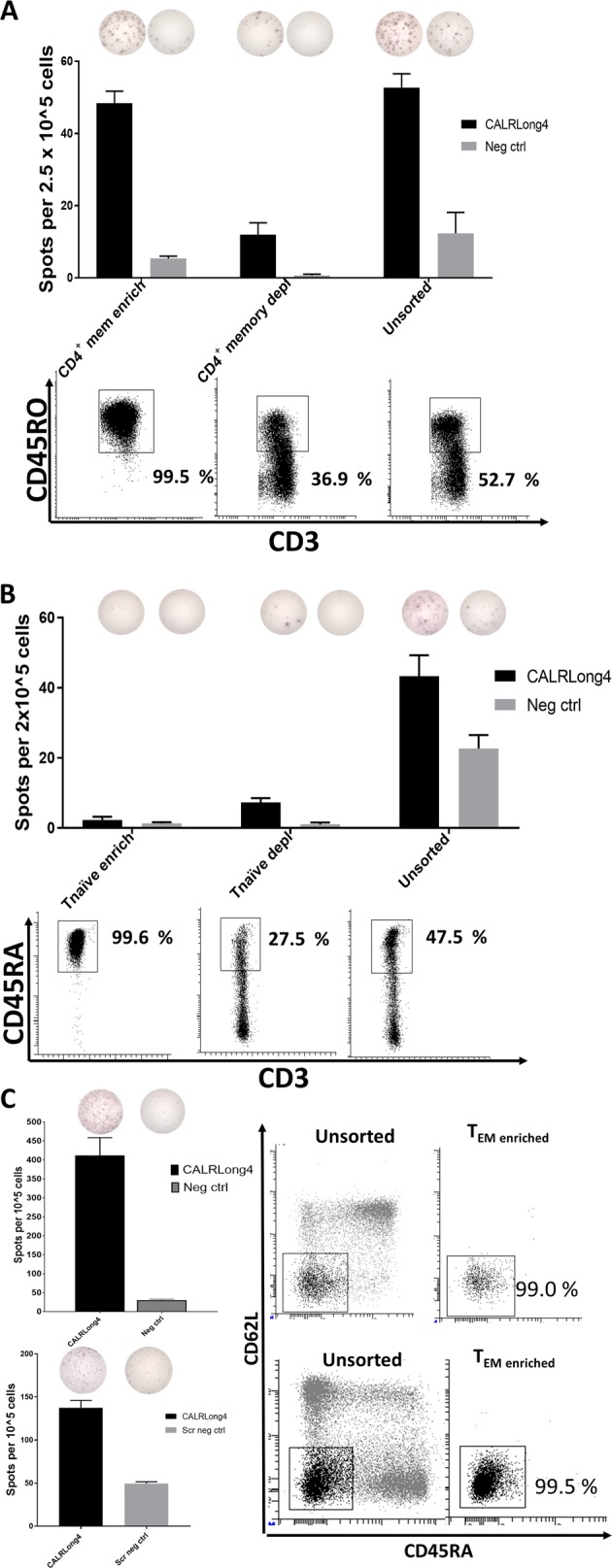
*CALR*-mutant-specific T-cell responses in different T-cell compartments. **a** PBMCs from healthy individuals were enriched for CD4^+^ T_mem_ with MACS. Enriched cells, CD4^+^ T_mem_-depleted cells, and unsorted cells were analyzed in a standard IFN-γ ELISPOT. (*Middle*) The number of spots per T-cell fraction and (*top*) a representative photograph of wells stimulated with either CALRLong4 or a negative scrambled peptide. (*Bottom*) Purity analyses of CD3^+^ gated cells in corresponding cell fractions. ELISPOT assays were performed in triplicate. **b** PBMCs from healthy individuals were enriched for T_naive_ cells with MACS. Enriched cells, T_naive_-depleted cells, and unsorted cells were analyzed in a standard IFN-γ ELISPOT. (*Middle*) The number of spots per T-cell fraction; (*top*) a representative photograph of wells stimulated with either CALRLong4 or a negative scrambled peptide. (*Bottom*) Purity analyses of CD3^+^ gated cells in corresponding cell fractions. ELISPOT assays were performed in triplicates. **c** PBMCs from healthy individuals were enriched for T effector memory cells (T_EM_) (CD3^+^, CD45RA^-^, CD62L^−^) with FACS, and analyzed in an ex vivo IFN-γ ELISPOT. (*Left*) The number of spot-forming cells and a representative photograph of wells stimulated with either CALRLong4 or a negative scrambled peptide. (*Right*) FACS plots of unsorted PBMCs and T_EM_-enriched fractions show the purity of T_EM_-enriched fractions in CD3^+^ gated cells. Error bars display standard error of the mean

Given the high frequencies and amplitudes of *CALR*-mutant-specific T-cell responses in healthy individuals, we speculated that these T-cell responses might be attributable to T_mem_ rather than T_naive_. To investigate this hypothesis, we isolated CD4^+^ T_mem_ from a healthy individual that had shown a clear CALRLong4-specific T-cell response. In these CD4^+^ T_mem_ enriched cells, we readily detected a strong in vitro immune response against CALRLong4 (Fig. 4a). Similarly, we isolated enriched T_naive_ from the same donor, and these cells did not show a specific response to CALRLong4 in vitro (Fig. 4b). However, we detected a CALRLong4-specific immune response in the unsorted PBMC fraction and in the T_naive_-depleted fraction. Next, we used FACS to enrich PBMC from two healthy donors for T effector memory T-cells (T_EM_), which were stimulated ex vivo in ELISPOT with CALRLong4. Strong ex vivo immune responses in the highly pure T_EM_ cell fractions were detected (Fig. 4c).

### Ex vivo CALR-mutant-specific immune responses reside primarily in the CD4^+^ T_mem_ compartment

**Fig. 5:**
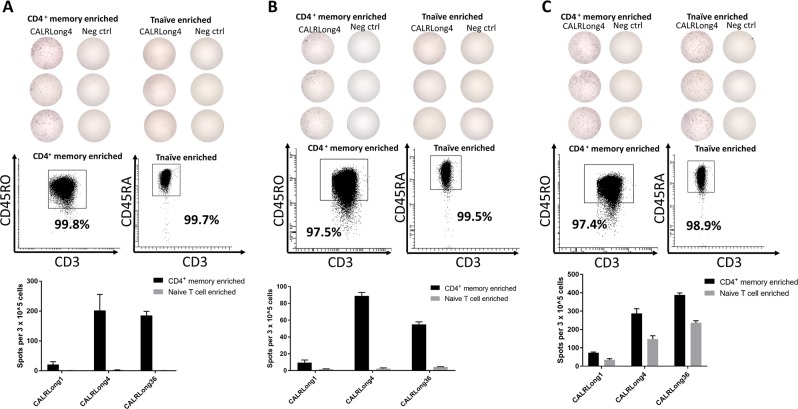
*CALR*-mutant-specific ex vivo responses in both CD4^+^ T_mem_ and T_naive_ compartments. PBMCs from three healthy individuals (**a**, **b**, and **c**) were enriched for CD4^+^ T_mem_ and T_naive_ with MACS. Enriched cells were analyzed in an ex vivo IFN-γ ELISPOT. (*Top*) Photographs of wells stimulated with either CALRLong4 or a negative scrambled peptide; (*middle*) analysis of purity of the different cell fractions in CD3^+^ gated cells; (*bottom*) graph shows the number of cells specific for CALRLong1, CALRLong4, and CALRLong36. The number of peptide-specific cells was calculated by subtracting the number of spots in wells stimulated with a scrambled negative control peptide from the number of spots in peptide-stimulated wells. Error bars display standard error of the mean

As the use of FACS for sorting T_EM_ cells can potentially interfere with cell functionality, we looked further into the CALR-mutant-specific T-cell responses by sorting PBMCs from three healthy individuals using MACS to enrich for CD4^+^ T_mem_ and T_naive_. The enrichments were based on negative selection; i.e., the cells of interest were not treated with antibodies that could either activate or inactivate the cells. We investigated the enriched T cells with an ex vivo IFN-γ ELISPOT assay for responses against CALRLong1, CALRLong4, and CALRLong36. The CD4^+^ T_mem_ from all donors displayed strong *CALR*-mutant-specific T-cell responses, mainly against CALRLong4 and CALRLong36. In samples from two individuals, the enriched T_naive_ fraction did not display any immune responses (Fig. 5a, b). However, in one individual, both the CD4^+^ T_mem_ fraction and the T_naive_ fraction displayed responses against the three mutant epitopes (Fig. 5c). However, the strongest immune response was identified in the T_mem_ fraction.

**Fig. 6:**
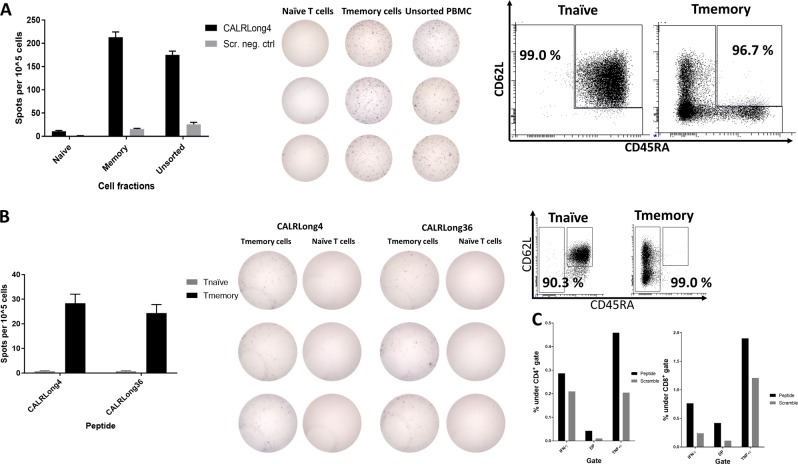
*CALR*-mutant-specific responses in FACS-sorted T_mem_, but not in T_naive_. **a** PBMCs from a healthy individual were enriched for T_mem_ (CD3^+^, CD62L^+^, CD45RA^−^ or CD3^+^, CD62L^−^, CD45RA^+^ or CD3^+^, CD62L^−^, CD45RA^−^) and T_naive_ (CD3^+^, CD62L^+^, CD45RA^+^). Cells were analyzed in an ex vivo IFN-γ ELISPOT to determine the numbers of spot-forming cells (*left*) in each cell fraction that responded to CALRLong4. (*Middle*) photographs of wells. (*Right*) Analysis of purity of the different T-cell fractions in CD3^+^ gated cells. **b** PBMCs isolated from freshly drawn blood from an older healthy individual with a clear CALRLong4-specific response were rested overnight in an incubator. Cells were sorted into T_mem_ and T_naive_, and analyzed for peptide-specific T-cell responses in an ex vivo IFN-γ ELISPOT assay. The number of spot-forming cells was determined in each cell fraction for cells stimulated with (*left*) CALRLong4 or (*middle*) CALRLong36. (*Right*) FACS purity analysis results. Due to difficulties in separating T_naive_ (CD3^+^, CD62L^+^, CD45RA^+^) and T_EMRA_ (CD3^+^, CD62L^-^, CD45RA^+^) cells, the sorting gates were set differently from those set in **a**, to avoid contamination of T_naive_ and T_EM_ enrichments. **c** T_mem_ isolated in **b** were intracellularly stained after one week of in vitro stimulation with the CALRLong4 peptide. Panel shows the cytokines produced by (*left*) CD4^+^ T-cells and (*right*) CD8^+^ T-cells upon stimulation with CALRLong4 or a negative control scrambled peptide. Error bars display standard error of the mean

To clarify whether these results might be reproducible by using another enrichment procedure, we performed FACS live-cell sorting to isolate either T_mem_ (CD3^+^, CD62L^+^, CD45RA^-^; CD3^+^, CD62L^−^, CD45RA^+^; and CD3^+^, CD62L^-^, CD45RA^−^) or T_naive_ (CD3^+^, CD62L^+^, CD45RA^+^) fractions. Next, we analyzed the enriched fractions and the unsorted cells using ex vivo IFN-γ ELISPOT to identify spontaneous immune responses against CALRLong4. Once more, we detected responses in enriched T_mem_, whereas no significant responses were detected in the T_naive_ fraction (Fig. 6a). To determine whether the cryopreservation of PBMC might have influenced the responses, we isolated PBMC from freshly drawn blood from a healthy individual with a known response to CALRLong4. The PBMC were rested overnight and then live-sorted with FACS and assayed in an ex vivo IFN-γ ELISPOT. Upon stimulation with CALRLong4 and CALRLong36, we once more detected cytokine release from T_mem_, but not from T_naive_ (Fig. 6b). Next, T_mem_ from the cell sorting were stimulated in vitro for 7 days with the CALRLong4 peptide and analyzed with ICS to investigate the phenotype of the responding T_mem_. Accordingly, we demonstrated that both CD4^+^ and CD8^+^ T_mem_ responded to stimulation with CALRLong4 (Fig. 6c). Due to the low yield of T_naive_ from the cell sorting procedure, we did not analyze potential CALRLong4-specific responses in T_naive_ with ICS.

### *CALR*-mutant-specific immune responses reside primarily in the T_EM_ and T_CM_ fractions

**Fig. 7:**
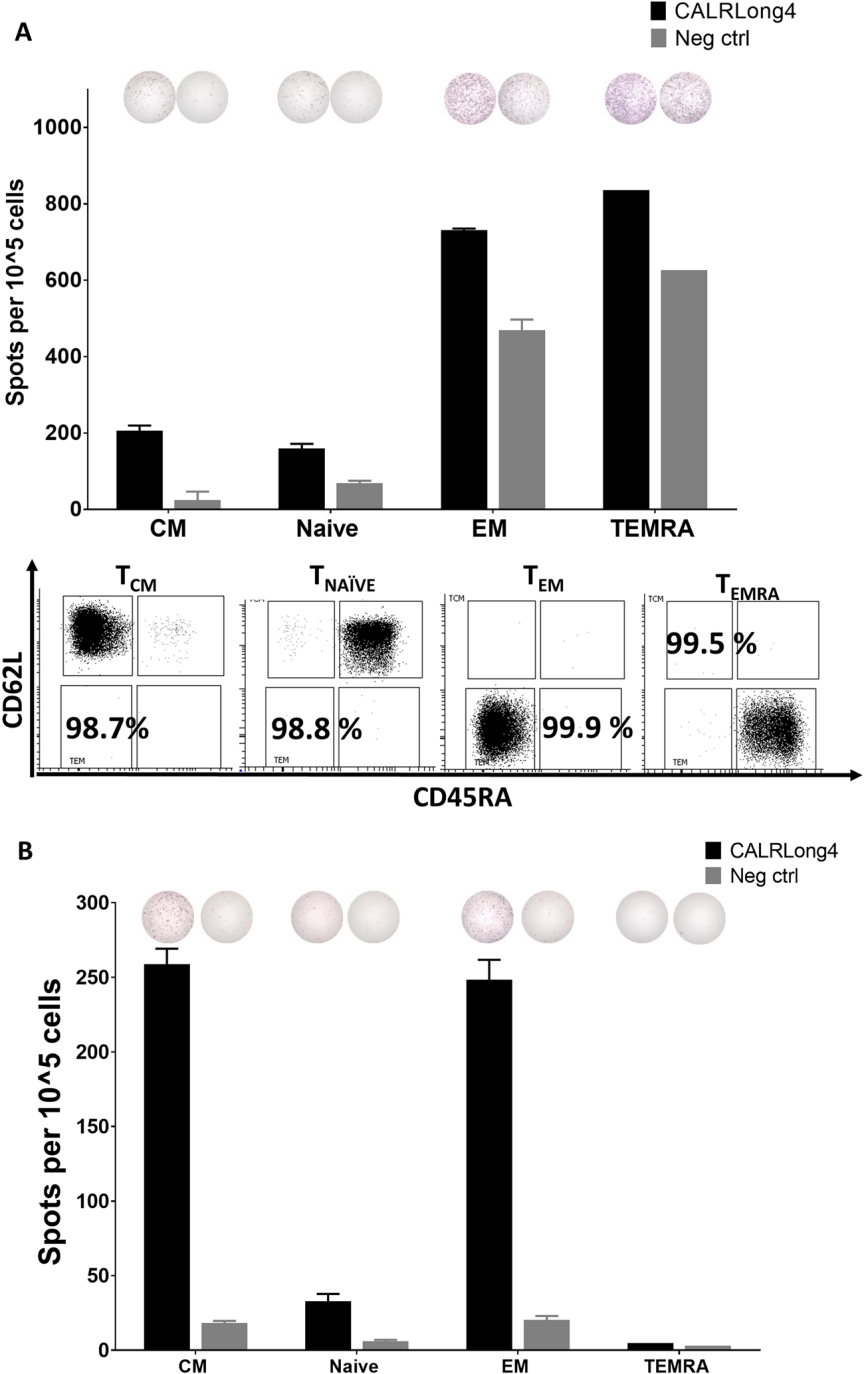
*CALR*-mutant-specific responses in both T_EM_ and T_CM_. **a** PBMCs from a healthy individual were enriched for T effector memory cells (T_EM_: CD3^+^, CD62L^-^ CD45RA^−^), T central memory cells (T_CM_: CD3^+^, CD62L^+^, CD45RA^-^), T_EMRA_ (CD3^+^, CD62L^−^, CD45RA^+^), and T_naive_ (CD3^+^, CD62L^+^, CD45RA^+^). Cells were analyzed in an ex vivo IFN-γ ELISPOT for responses against CALRLong4. (*Top*) Representative wells for each cell fraction show responses to both CALRLong4 and a scrambled negative control peptide; (*middle*) graph shows the numbers of spot-forming cells in each cell fraction. (*Bottom*) Analysis of purity of the different T-cell fractions in CD3^+^ gated cells. Due to cell scarcity, T_EM_ and T_EMRA_ responses were measured in duplicate and in a single experiment, respectively. T_CM_ and T_näive_ responses were measured in triplicate. **b** PBMCs from healthy individuals were sorted and analyzed as described above. (*Top*) Representative wells for each cell fraction show responses to both CALRLong4 and a scrambled negative control peptide. (*Bottom*) Graph shows the number of spot-forming cells in each cell fraction. Due to cell scarcity, we did not perform purity analyses. T_EMRA_ responses were analyzed in a single experiment, but all other cell fractions were analyzed in triplicate. Error bars display standard error of the mean

As the CALR-mutant specific T cells clearly reside within the T_mem_ compartment we then examined which of the cells in this compartment are the most reactive to the *CALR*-mutations. Hence, we used FACS live-cell sorting to isolate four different CD3^+^ T-cell fractions: T_EM_ (CD3^+^, CD62L^−^, CD45RA^−^), T central memory cells (T_CM_: CD3^+^, CD62L^+^, CD45RA^−^), terminally differentiated memory cells (T_EMRA_: CD3^+^, CD62L^−^, CD45RA^+^), and T_naive_ (CD3^+^, CD62L^+^, CD45RA^+^). Then, we analyzed CALRLong4-specific immune responses in sorted cells using ex vivo IFN-γ ELISPOT assays. Cells from two different individuals were analyzed. The first sample showed the highest antigen-specific immune responses in the T_EM_ and T_EMRA_ fractions (Fig. 7a, top). However, due to the scarcity of cells, we only analyzed T_EM_ cell responses in duplicate wells and T_EMRA_ cell responses in a single well. We also identified some reactivity in the T_CM_ fraction. The lowest cytokine release was identified in the T_naive_ fraction. Purity analyses of the enriched fractions invariably showed very high purity (Fig. 7a, bottom). Repeating the sorting procedure in another donor we got a higher cell yield and were able to analyze T_EM_, T_CM_, and T_naive_ fractions in triplicate experiments. Both the T_CM_ and T_EM_ fractions of this sample displayed very strong CALRLong4-specific immune responses. In contrast, the T_naive_ and T_EMRA_ responses were very weak (Fig. 7b).

### CALR-mutant specific T-cell cultures from CD4^+^ T_mem_ recognize several epitopes from the mutant CALR C-terminus and upregulate CD107a upon antigen stimulation

**Fig. 8:**
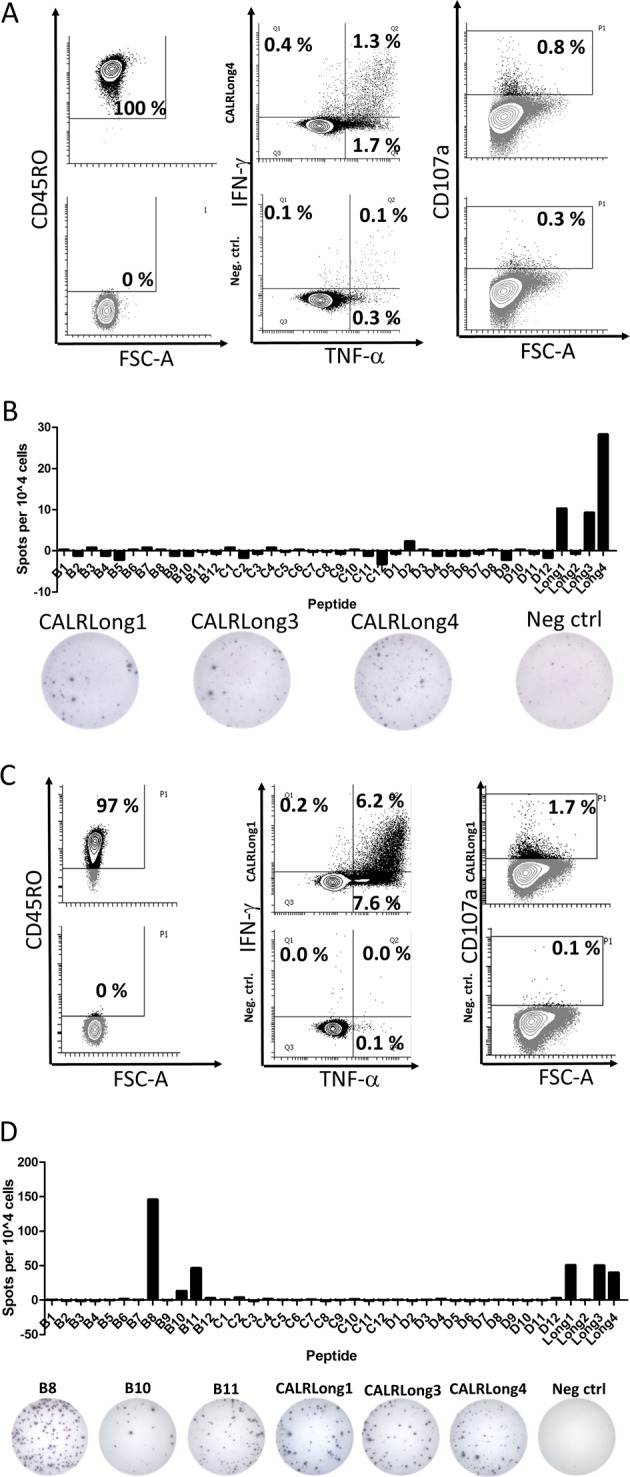
Establishment of two T-cell cultures specific for mutant CALR epitopes. **a** (*Left)* Purity analysis of isolated CD4^+^ T-memory cells with cells stained for CD45RO (*top, left*) and cells that were unstained (*below, left*). The percentages depict the amount of CD45RO^+^ cells from the CD3^+^ fraction. After three stimulations with autologous DC cells were analyzed for cytokine release against CALRLong4 (*top, middle*) and scrambled negative control peptide *(below, middle*). Cells were also analyzed for expression of CD107a upon stimulation with CALRLong4 (*top, right*) and negative control peptide (*below, right*). **b** The T-cell culture was analyzed for responses against the CALR library and several other CALR-mutant epitopes with the amount of spots shown (*top*), and representative wells displayed (*below*). **c** CD4^+^ T-memory cells were isolated from another donor and used to establish a T-cell culture specific for the CALRLong1 epitope. Purity analysis (*left*), cytokine release (*middle*), and CD107a upregulation were analyzed as in **a**. **d** The CALRLong1 specific T-cell culture was analyzed for responses against the CALR library. We identified responses against several short epitopes from the CALR library as well as long epitopes (*top*). Representative wells are shown (*below*)

We have previously demonstrated that it is possible to isolate and expand CALR-mutant specific T cells from *CALR*-mutant MPN-patients. To further verify, that CALR-mutant specific T cells in healthy donors are indeed antigen experienced T_mem_, we chose to analyze whether it is possible to isolate and expand CALR-mutant specific T cells from the T_mem_ compartment of healthy donors. Hence, using MACS we enriched CD4^+^ T_mem_ from PBMC from two different healthy donors. The cells were enriched twice to ensure a high purity of cells (Fig. 8a, left and 8c, left). Next, the CD4^+^ T_mem_ were stimulated three times with autologous DCs that were either pulsed with CALRLong1 or CALRLong4 peptide. Antigen responses were measured at day 5 after each DC stimulation using ICS. A total of three DC stimulations were performed. Due to scarcity of T cells, the CALRLong1 specific culture was expanded after the third DC stimulation using our rapid expansion protocol with high dose IL-2. The two T-cell cultures responded with cytokine release (Fig. 8a, c, middle panel), and enhanced expression of the degranulation marker CD107a upon stimulation with antigen (Fig. 8a, c, right panel). This last finding is especially noteworthy, as CALR-mutant specific T cells from patients were shown to display specific cytotoxicity to autologous *CALR*-mutant cells through upregulation of CD107a^4^. Finally, by analyzing immune responses from the two T-cell cultures, we demonstrated that the CALR-mutant specific T cells recognize several epitopes in the mutant C-terminus (Fig. 8b, d). Thus, antigen experienced T cells isolated and expanded from healthy donors do secrete cytokines upon stimulation with several epitopes from the mutant CALR C-terminus, and these T cells are potentially able to kill *CALR*-mutant cells.

## Discussion

We previously described in detail that the immune system of *CALR*-mutant patients specifically reacts to the mutant CALR C-terminus^3^. Even more, we showed that CALR-mutant specific T cells cloned from *CALR*-mutant patients recognize and kill autologous *CALR*-mutant target cells in a *CALR*-mutant-dependent manner^4^. However, in *CALR*wt individuals, the immune system should not have been challenged with mutant CALR epitopes, and accordingly such individuals should not display a CALR-mutant specific immune response. Surprisingly, we identified CALR-mutant specific immune responses in several *JAK2*V617F-mutant MPN-patients. Although we did not test whether these patients also carried *CALR* mutations, we speculated that it would be highly unlikely as *JAK2* and *CALR* double mutants are rare^14–16^. This finding lead us to analyze CALR-mutant specific responses in healthy donors, and we were surprised to find even stronger and more frequent responses in healthy donors compared to the responses identified by us in *CALR*-mutant MPN-patients^3^. The stronger and more frequent responses might be explained by the theory of cancer immuno-editing;^23^ cancer cells may be cleared by the immune system (in healthy individuals), be held in equilibrium by the immune system (in the MPN-setting; patients with essential thrombocythemia), or escape the immune system and enter into the metastatic disseminated stage (in the MPN-setting; patients with myelofibrosis)^23,24^. By following this notion, the data described here implies, that healthy individuals are able to clear cells harboring the *CALR*-mutation and, accordingly, this tumor cell elimination generates CALR-mutant specific T_mem_. Interestingly, Tubb et al.^25^ recently isolated CALR-mutant specific CD8^+^ T cells from peripheral blood in healthy donors using HLA-I tetramers. Even though this study did not provide data on the frequency of healthy donors harboring CALR-mutant specific T cells, and, most importantly, did not investigate the phenotype of such T cells, it still supports our findings of immune responses against mutant CALR in healthy individuals.

As such, no studies have investigated the phenotype of neo-antigen-specific T cells in healthy donors, and only a limited number of studies have investigated the memory phenotype of T cells that display reactivity to tumor-associated antigens. Pittet et al.^26^ investigated the phenotype of CD8^+^ T cells from healthy individuals with a reactivity against the melanoma associated antigen MART-1 and found the specific T cells to be T_naive_. In contrary we describe by several different means, that T_mem_ are activated both in vitro and ex vivo upon stimulation with the mutant CALR antigen. Importantly, we further describe that T-cell cultures derived from high purity T_mem_ fractions from two healthy donors reacted against several epitopes in the mutant CALR C-terminal. The data presented in this study imply that the immune system in healthy individuals has been exposed to *CALR*-mutant cells. Given the exquisite immunogenicity of these mutations, the *CALR*-mutant cells have been cleared by the immune system before the establishment of overt malignancy and, consequently T-cell memory to the *CALR*-mutations has been generated.

We did not only detect frequent and strong T-cell responses among in vitro stimulated T cells, but also directly ex vivo. These ex vivo responses are especially remarkable as it, even in cancer patients, is rare to detect tumor-associated antigen-specific T-cells ex vivo by tetramer staining or ELISPOT^27^. As such, it is unusual for cells from healthy individuals to display a high frequency of specific immunity against a cancer neo-antigen.

Given the high frequency of responses to the mutant antigens we verified that the CALR mutations do not share sequence homology with any other known epitope. We also ordered peptides from three different peptide providers (see Materials and methods) in order to rule out the possibility of peptide impurities as an explanation to the T-cell responses. Furthermore, the established T-cell cultures derived from T_mem_ recognized several epitopes in the mutant CALR C-terminus, demonstrating that the observed responses indeed are CALR-mutant specific T-cell responses.

The high frequency of CALR-mutant-specific immune responses observed in healthy individuals samples raises the question why some individuals develop *CALR*-mutant MPN. The functionality of the immune system is believed to decline with age, and the median age at diagnosis of *CALR*-mutant MPN is reported to be 47–57 years^16,19–22^. Therefore, we examined the frequency of immune responses to the CALR-mutant C-terminus in two age defined cohorts (median ages: 19 years vs. age 57 years). However, no differences were found in neither frequency nor response between to two age defined cohorts.

As the *CALR* mutations are highly immunogenic it might seem paradoxical, that the disease is not cleared by the immune system. However, there are several explanations to the paradoxical emergence of *CALR*-mutant MPN. Firstly, it was recently shown, that *CALR*-mutant patients display lower levels of human leukocyte antigen-I^28^, and patients with MPN show increased levels of myeloid derived suppressor cells^29^. Although these explanations could partially clarify why *CALR*-mutant cells are able to evade immune-mediated destruction, it does not explain why *CALR*-mutant cells are initially able to establish themselves in the bone marrow, evade immune-mediated destruction, expand and develop overt *CALR*-mutant MPN. We are currently investigating a cohort of healthy individuals for low-burden *CALR*-mutations and hope that we thereby may identify healthy individuals with low-burden *CALR*-mutation. A thorough analysis of the immune constitution of such individuals might add important new information on the development and evolution of *CALR*-mutant MPN, which could potentially be prevented by therapeutic cancer vaccination with CALR-mutant epitopes.

In conclusion, we have demonstrated that a majority of healthy individuals harbor immune cells that display CALR-mutant-specific T-cell responses, and that the frequency and amplitude of these responses are higher than in *CALR*-mutant patients. Neither do the frequency nor amplitude of responses depend on age. Moreover, we show that the majority of the immune responses are attributed to T_mem_, particularly cells of the T_EM_ and T_CM_ compartments. In essence this demonstrates that the immune system in healthy individuals has eliminated tumor cells and generated T-cell memory, thus demonstrating the first of the three E’s - elimination - in the theory of cancer immuno-surveillance/editing^24^.

## Supplementary information

Supplementary Material 1

Supplementary Material 2

Supplementary

Supplementary Material 4

Supplementary Material 5

Supplementary Material 6

Supplementary materials legends
