# Supplementary material for: High frequencies of circulating memory T cells specific for calreticulin exon 9 mutations in healthy individuals
Source: Blood Cancer J. 2019 Jan 17;9(2):8. doi: 10.1038/s41408-018-0166-4 (PMC6336769; doi:10.1038/s41408-018-0166-4)
Supplement: Supplementary file 1 — Supplementary Material 1 [file 41408_2018_166_MOESM1_ESM.pptx]

## Slide 1
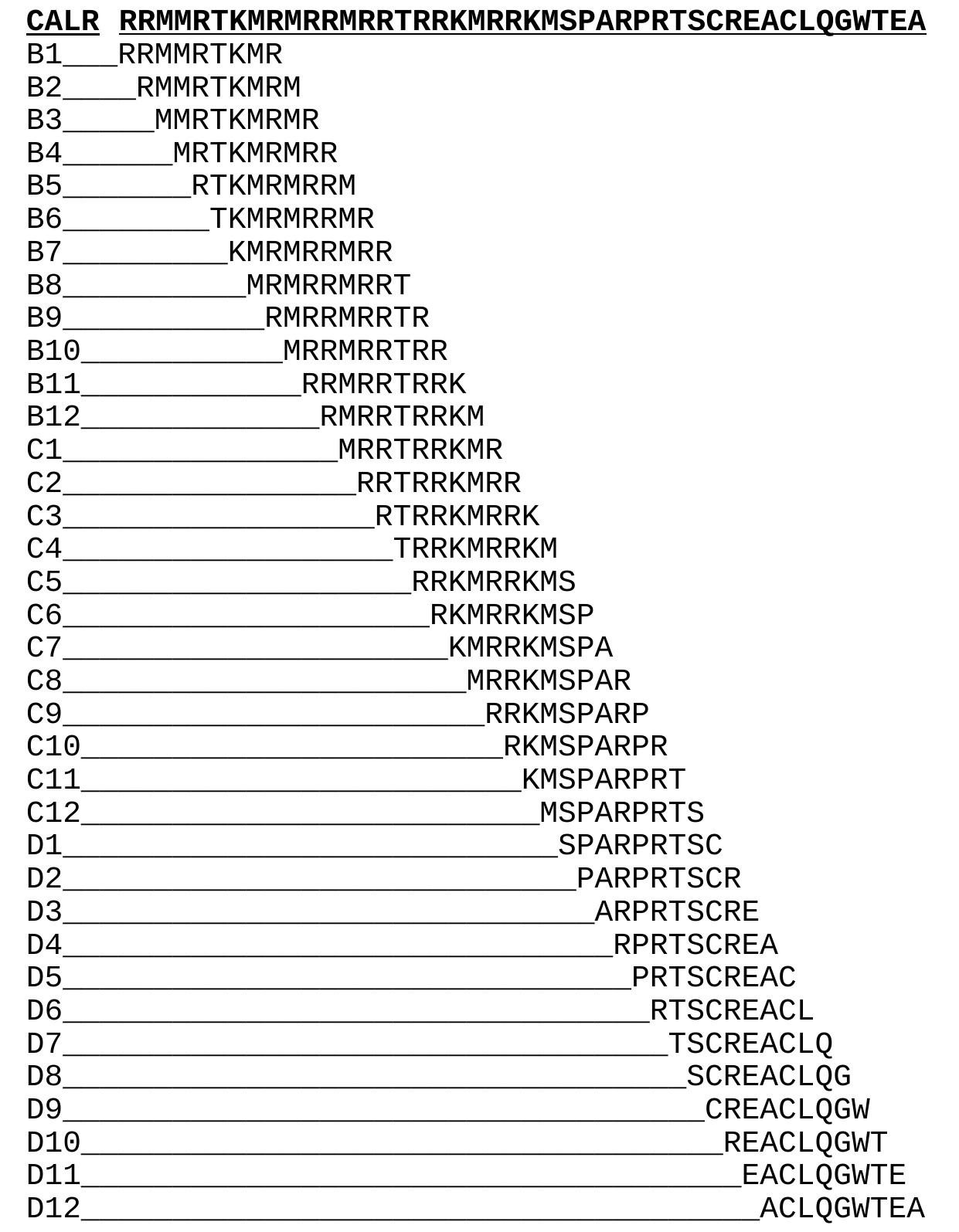

RRMMRTKMRMRRMRRTRRKMRRKMSPARPRTSCREACLQGWTEA
# CALR B1___RRMMRTKMR B2____RMMRTKMRM B3_____MMRTKMRMR B4______MRTKMRMRR B5_______RTKMRMRRM B6________TKMRMRRMR B7_________KMRMRRMRR B8__________MRMRRMRRT B9___________RMRRMRRTR B10___________MRRMRRTRR B11____________RRMRRTRRK B12_____________RMRRTRRKM C1_______________MRRTRRKMR C2________________RRTRRKMRR C3_________________RTRRKMRRK C4__________________TRRKMRRKM C5___________________RRKMRRKMS C6____________________RKMRRKMSP C7_____________________KMRRKMSPA C8______________________MRRKMSPAR C9_______________________RRKMSPARP C10_______________________RKMSPARPR C11________________________KMSPARPRT C12_________________________MSPARPRTS D1___________________________SPARPRTSC D2____________________________PARPRTSCR D3_____________________________ARPRTSCRE D4______________________________RPRTSCREA D5_______________________________PRTSCREAC D6________________________________RTSCREACL D7_________________________________TSCREACLQ D8__________________________________SCREACLQG D9___________________________________CREACLQGW D10___________________________________REACLQGWT D11____________________________________EACLQGWTE D12_____________________________________ACLQGWTEA
