# Supplementary figures and images for: High frequencies of circulating memory T cells specific for calreticulin exon 9 mutations in healthy individuals
Source: Blood Cancer J. 2019 Jan 17;9(2):8. doi: 10.1038/s41408-018-0166-4 (PMC6336769; doi:10.1038/s41408-018-0166-4)

## Slide 1
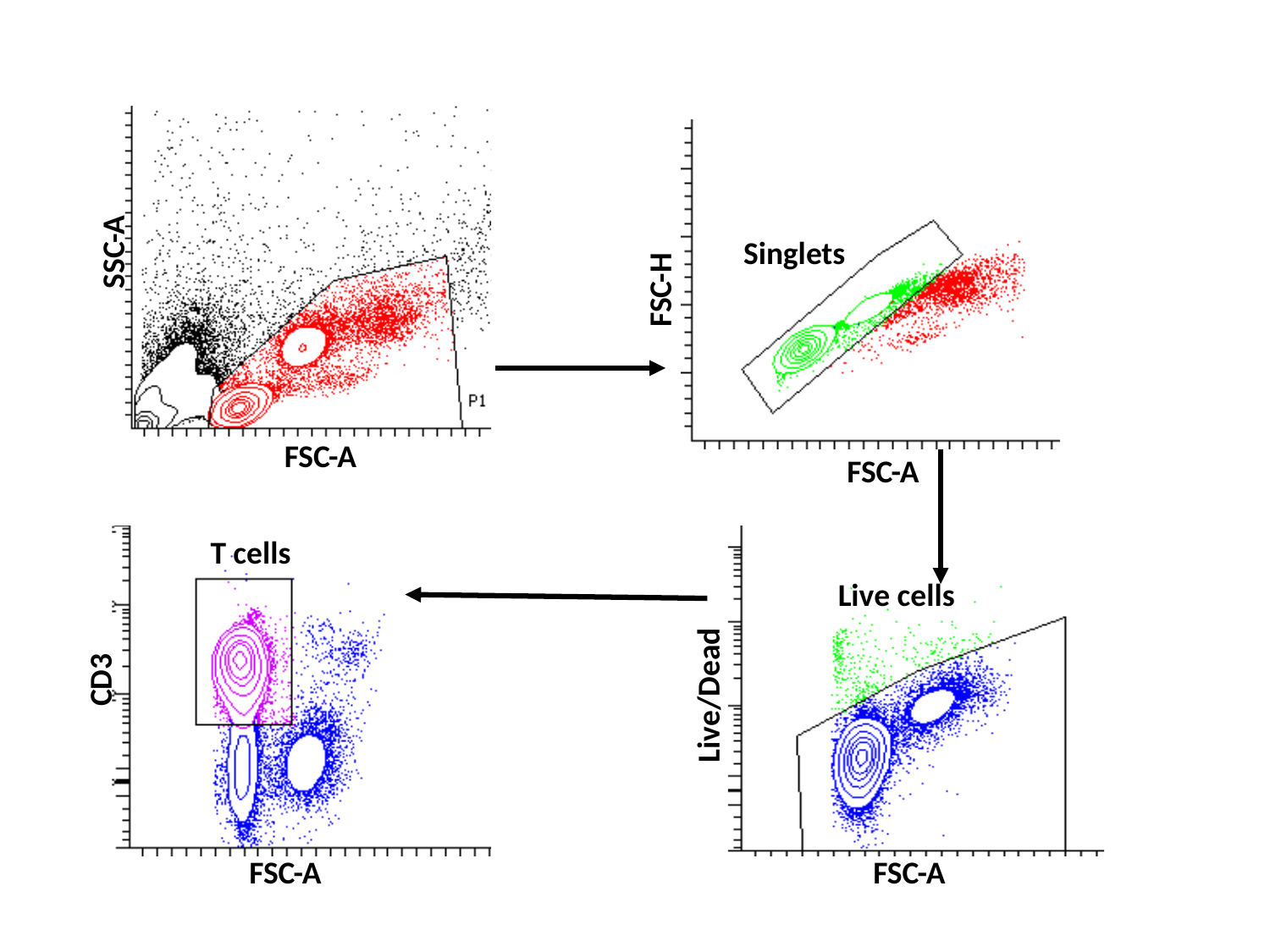

SSC-A
Singlets
FSC-H
FSC-A
FSC-A
T cells
Live cells
CD3
Live/Dead
FSC-A
FSC-A

Supplement: Supplementary file 2 — Supplementary Material 2 [file 41408_2018_166_MOESM2_ESM.pptx]

## Slide 1
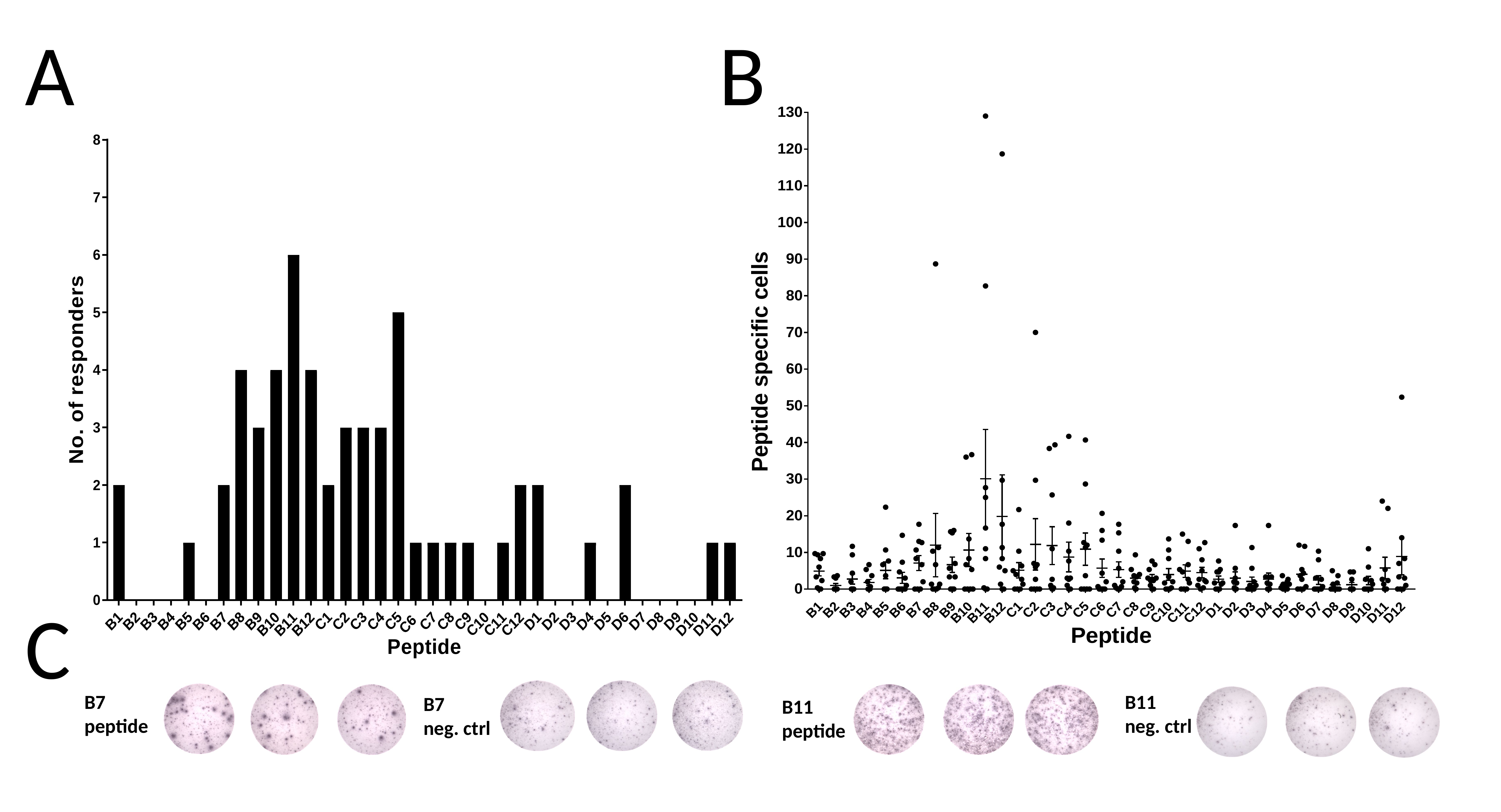

A
B
C
B7 peptide
B11
neg. ctrl
B7
neg. ctrl
B11
peptide

Supplement: Supplementary file 4 — Supplementary Material 4 [file 41408_2018_166_MOESM4_ESM.pptx]

## Slide 1
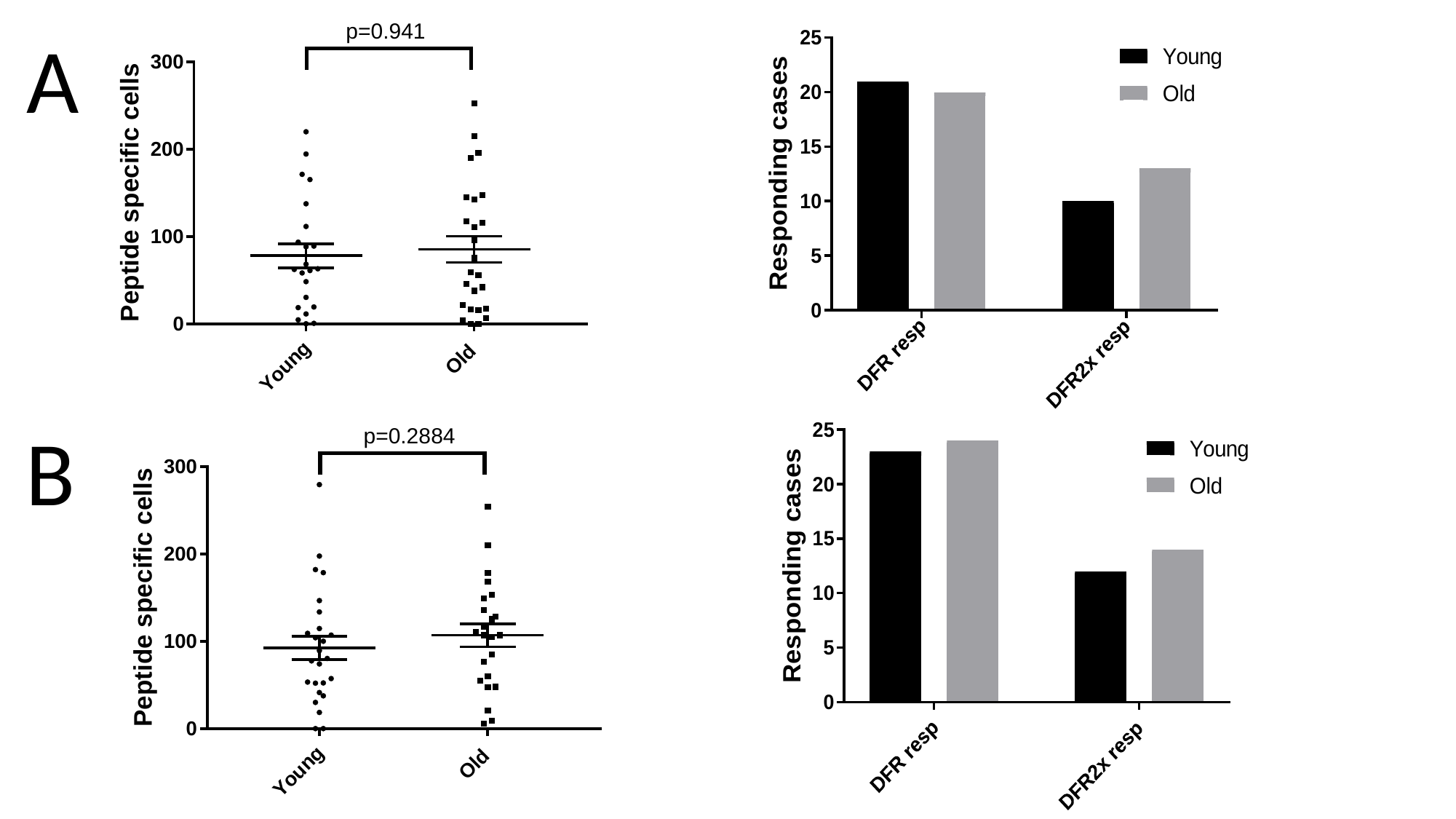

A
B

Supplement: Supplementary file 5 — Supplementary Material 5 [file 41408_2018_166_MOESM5_ESM.pptx]
