# Supplementary material for: High frequencies of circulating memory T cells specific for calreticulin exon 9 mutations in healthy individuals
Source: Blood Cancer J. 2019 Jan 17;9(2):8. doi: 10.1038/s41408-018-0166-4 (PMC6336769; doi:10.1038/s41408-018-0166-4)
Supplement: Supplementary file 3 — Supplementary [file 41408_2018_166_MOESM3_ESM.docx]

**Supplementary material and methods**

**Intracellular cytokine staining (ICS)**

ICS was performed as previously described.^1^ We used the following fluorochrome-tagged antibodies: CD3-FITC or APC-H7, CD4-PerCP or FITC (both BD Biosciences, San José, CA, USA), CD8-Pacific Blue or PerCP (BioLegend, San Diego, CA, USA), IFN-γ-PE or PE-Cy7, TNF-α-APC BV421 and CD107a-PE (BD Biosciences, San José, CA, USA). Dead cells were stained with the LIVE/DEAD Fixable Near-IR Dead Cell Stain Kit (Waltham, MA, USA) or with Fixable Viability Stain 510 (BD Biosciences, San José, CA, USA). All ICS experiments were performed in triplicates, with 3×10^5^ cells/well. After the indicated incubation times, cells were pooled into one well and stained together. Gating strategy for the identification of CD4^+^ and CD8^+^ T-cells is provided in Supplementary Material 6.

**Establishment of T cell cultures specific for mutant CALR epitopes**

Dendritic cells from healthy donors were manufactured as previously described.^1^ One day prior to stimulation with peptide pulsed DC, the monocyte depleted cell fraction was enriched for CD4^+^ memory T cells using MACS as described above. The memory cells were allowed to rest overnight in incubator and were then stimulated with peptide pulsed dendritic cells the next day. Two days after DC stimulation the memory cells were stimulated with IL-2 (120 U/mL). A total of three DC stimulations were performed.

**Statistical analysis**

We performed statistical analyses of ELISPOT responses with the distribution free resampling (DFR) method and with the more conservative, DFR2x method, described by Moodie et al.^2^ P-values ≤0.05 were deemed significant. All continuous variables were analyzed with the Mann-Whitney U-test, performed with the statistical software package, Prism v. 7 (GraphPad Software Inc., San Diego, CA, USA).

**References**

1 Holmstrom MO, Martinenaite E, Ahmad SM, Met O, Friese C, Kjaer L *et al.* The calreticulin (CALR) exon 9 mutations are promising targets for cancer immune therapy. *Leukemia* 2018; **32**: 429–437.

2 Moodie Z, Price L, Gouttefangeas C, Mander a., Janetzki S, Löwer M *et al.* Response definition criteria for ELISPOT assays revisited. *Cancer Immunol Immunother* 2010; **59**: 1489–1501.
