# Supplementary material for: High frequencies of circulating memory T cells specific for calreticulin exon 9 mutations in healthy individuals
Source: Blood Cancer J. 2019 Jan 17;9(2):8. doi: 10.1038/s41408-018-0166-4 (PMC6336769; doi:10.1038/s41408-018-0166-4)
Supplement: Supplementary file 6 — Supplementary Material 6 [file 41408_2018_166_MOESM6_ESM.pptx]

## Slide 1
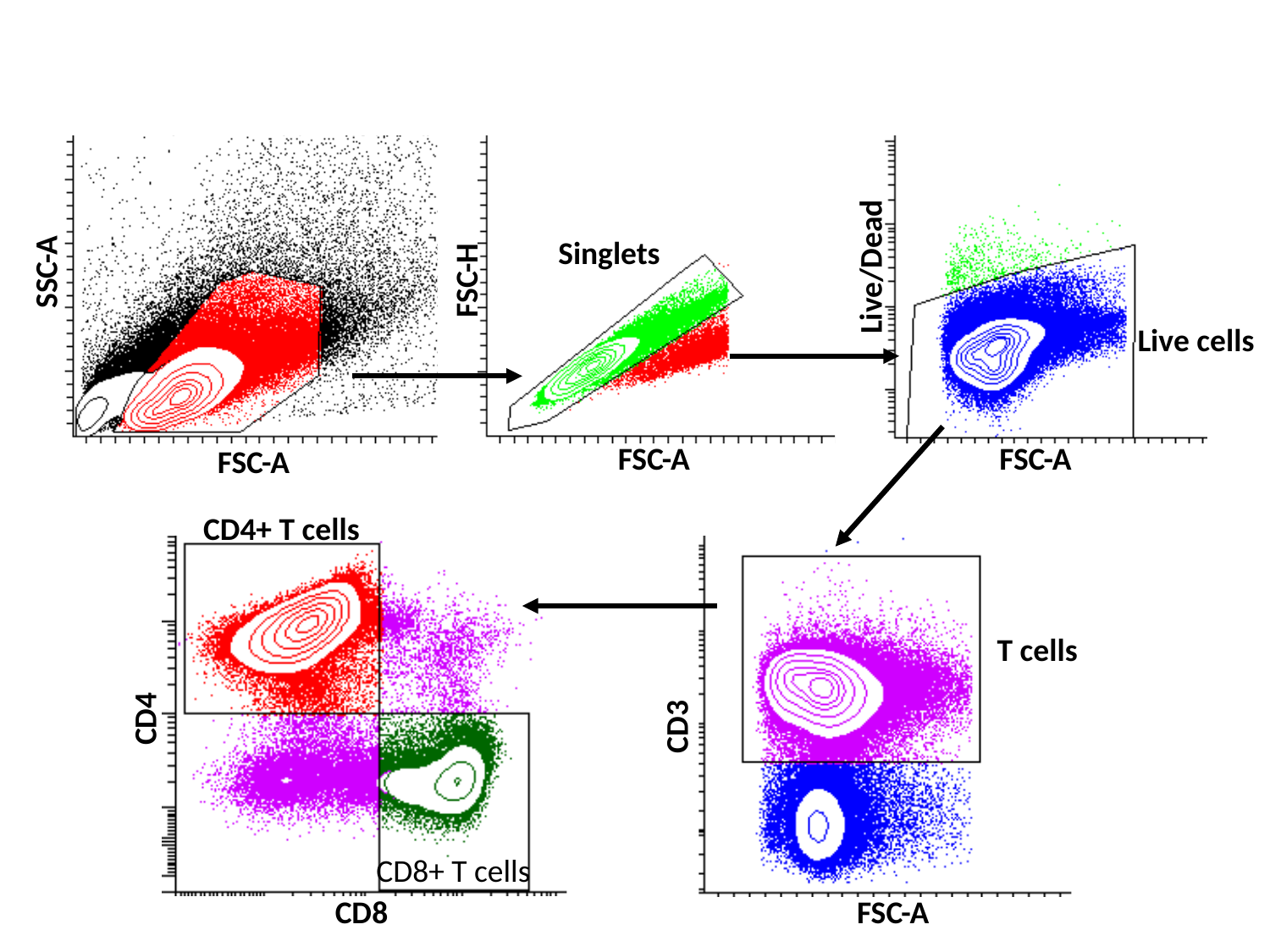

SSC-A
Singlets
FSC-H
Live/Dead
Live cells
FSC-A
FSC-A
FSC-A
CD4+ T cells
T cells
CD4
CD3
CD8+ T cells
CD8
FSC-A
