# Supplementary material for: High frequencies of circulating memory T cells specific for calreticulin exon 9 mutations in healthy individuals
Source: Blood Cancer J. 2019 Jan 17;9(2):8. doi: 10.1038/s41408-018-0166-4 (PMC6336769; doi:10.1038/s41408-018-0166-4)
Supplement: Supplementary file 7 — Supplementary materials legends [file 41408_2018_166_MOESM7_ESM.docx]

**SUPPLEMENTARY MATERIAL LEGENDS**

**Supplementary Material 1:** Amino acid sequences of the 36 nonamer epitopes in the CALR-library.

**Supplementary Material 2:** Gating strategy used to isolate CD3^+^ T-cells for analysis of memory and naïve T-cell phenotype and for live cell sorting of different T memory cell subtypes.

**Supplementary Material 3: Supplementary Material and Methods.**

**Supplementary Material 4: Cells from 10 healthy individuals were analyzed for immune responses against 36 overlapping nonamer epitopes that covered the entire sequence of the CALR mutant C-terminus**. **A.** The frequency of DFR-defined ELISPOT responses to all peptides. **B.** Spot specific cells from each donor against each peptide. **C.** Examples of ELISPOT responses exhibited by PBMCs from healthy individuals against the nonamer CALR mutant epitopes, B7 (*left*) and B11 (*right*). Error bars display standard error of the mean.

**Supplementary Material 5: Cells from healthy donors from two age defined cohorts were analyzed for frequency and amplitude of responses to CALR-mutant epitopes. A.** (*Left*) Peptide-specific response against CALRLong1 in cells from young healthy individuals (median age 19 years) and cells from older healthy individuals (median age 57 years). (*Right*) Frequency of DFR- and DFR2x-defined CALRLong1-specific responses in cells from young and old healthy individuals. **B.** (*Left*) Peptide-specific responses against CALRLong4 in cells from young healthy individuals and cells from older healthy individuals. (*Right*) Frequency of DFR- and DFR2x-defined CALRLong4-specific responses in cells from young and older healthy individuals. Error bars display standard error of the mean.

**Supplementary Material 6:** Gating strategy for the gating of CD4^+^ and CD8^+^ T-cells for intracellular cytokine staining.
